# General Practitioners’ Experiences With Potentials and Pitfalls of Video Consultations in Norway During the COVID-19 Lockdown: Qualitative Analysis of Free-Text Survey Answers

**DOI:** 10.2196/45812

**Published:** 2023-03-20

**Authors:** Børge Lønnebakke Norberg, Linn Okkenhaug Getz, Tor Magne Johnsen, Bjarne Austad, Paolo Zanaboni

**Affiliations:** 1 General Practice Research Unit Department of Public Health and Nursing Norwegian University of Science and Technology Trondheim Norway; 2 Norwegian Centre for E-health Research Tromsø Norway

**Keywords:** general practice, video consultations, remote consultations, e-consultations, communication, patient safety, COVID-19, pandemic, lockdown, telehealth, telemedicine, healthcare, health care, user experience

## Abstract

**Background:**

The use of video consultations (VCs) in Norwegian general practice rapidly increased during the COVID-19 pandemic. During societal lockdowns, VCs were used for nearly all types of clinical problems, as in-person consultations were kept to a minimum.

**Objective:**

This study aimed to explore general practitioners’ (GPs’) experiences of potentials and pitfalls associated with the use of VCs during the first pandemic lockdown.

**Methods:**

Between April 14 and May 3, 2020, all regular Norwegian GPs (N=4858) were invited to answer a web-based survey, which included open-ended questions about their experiences with the advantages and pitfalls of VCs. A total of 2558 free-text answers were provided by 657 of the 1237 GPs who participated in the survey. The material was subjected to reflexive thematic analysis.

**Results:**

Four main themes were identified. First, VCs are described as being particularly convenient, informative, and effective for consultations with previously known patients. Second, strategically planned VCs may facilitate effective tailoring of clinical trajectories that optimize clinical workflow. VCs allow for an initial overview of the problem (triage), follow-up evaluation after an in-person consultation, provision of advice and information concerning test results and discharge notes, extension of sick leaves, and delivery of other medical certificates. VCs may, in certain situations, enhance the GPs’ insight in their patients’ relational and socioeconomical resources and vulnerabilities, and even facilitate relationship-building with patients in need of care who might otherwise be reluctant to seek help. Third, VCs are characterized by a demarcated communication style and the “one problem approach,” which may entail effectiveness in the short run. However, the web-based communication climate implies degradation of valuable nonverbal signals that are more evidently present in in-person consultations. Finally, overreliance on VCs may, in a longer perspective, undermine the establishment and maintenance of relational trust, with a negative impact on the quality of care and patient safety. Compensatory mechanisms include clarifying with the patient what the next step is, answering any questions and giving further advice on treatment if conditions do not improve or there is a need for follow-up. Participation of family members can also be helpful to improve reciprocal understanding and safety.

**Conclusions:**

The findings have relevance for future implementation of VCs and deserve further exploration under less stressful circumstances.

## Introduction

### Background

Until 2020, the use of video consultations (VCs) between general practitioners (GPs) and patients was less frequent and only slowly increased in both Norway and many other countries [1]. The COVID-19 pandemic resulted in a massive uptake of VCs, as a high number of GPs simultaneously adopted this modality over the course of a few weeks [2]. During the societal lockdown, VCs were used for nearly all types of clinical problems due to restrictions regarding the conduct of in-person consultations [2-4]. Moreover, most GPs were novices to VCs. The research-based knowledge on the use of VCs in general practice before 2020 was limited. Experiences with VCs that occurred after a nationwide large-scale adoption are important to understand this unprecedented transition period in general practice and for the future use of VCs between patients and GPs.

In the wake of the pandemic, VCs have come to represent a well-established consultation modality with a substantial knowledge base. More knowledge on potentials and pitfalls associated with VCs in different clinical and organizational contexts is, nevertheless, needed. For instance, the applicability of VCs might differ significantly between impersonal “drop-in” services and a regular GP scheme based on continuity of care. In a previous publication from the same lockdown period, regular GPs in Norway found VCs to be, in general, more suitable in the follow-up of previously presented health issues and known patients [4].

VCs represent an approximation of face-to-face interaction and a “visual upgrade” of telephone consultations [5,6]. Through VCs, patients have easy access to their GP without having to travel or take time off from work, and often without waiting [7-10]. High satisfaction with VCs has been reported among both patients and GPs [6]. Occasionally, VCs have been proven to improve patient care and safety [6,11]. However, VCs are not considered appropriate for all situations, and in-person consultations are, by many, considered the best alternative when possible [10]. VCs have been found to be more suitable where an existing doctor-patient relationship is established [4,5], as previously documented for in-person consultations [12-15]. While VCs might result in additional burden on GPs with an already busy schedule [16], they may also entail increased flexibility [4], which, in turn, can contribute to favorable changes in working practices [5]. Initial guidance has been attempted with regard to how and for which patients and health problems would VCs be useful or have unintended consequences [17,18].

In terms of communication, concerns regarding the quality of VCs have been raised [19]. Communication may be more demanding on video [20], and questions about responsibility, privacy, and safety may arise when patients are located in their home environment [21]. Differences in the level of digital literacy among patients might result in disparities in access [7,16]. Increased accessibility may lower the threshold for contacting the GP [22] and thereby reduce the GP’s time spent on other important tasks [16]. For instance, high availability of VCs might reduce other patients’ access to in-person consultations [23,24].

### Aim

The aim of this study was to explore regular GPs’ experiences of potentials and pitfalls with the use of VCs in Norway during a critical period with rapid uptake.

## Methods

### Overview

We conducted a web-based cross-sectional survey between April 14 and May 3, 2020, to explore the suitability of VCs among GPs in Norway during the COVID-19 lockdown. As described elsewhere [4], the survey was addressed to all GPs registered in the Norwegian regular GP scheme (Textbox 1) and conducted through the Netigate application. After answering a series of structured survey questions, participants were invited to answer 5 nonmandatory open-ended (free-text) questions about their personal experiences with VCs compared to face-to-face consultations with regard to (1) the advantages of VCs, (2) the pitfalls associated with VCs, (3) changes in working practices linked to the digital shift, (4) how VCs impact the GP’s role, and (5) other reflections. In total, 1237 GPs (26% of all GPs in Norway) responded to the main survey [4]. Among them, 657 GPs provided 1 or more free-text answers, 533 answered 2 questions, and 456 responded to all 5 questions. In total, 2558 free-text responses were obtained. The answers varied considerably in content and length.

**The regular general practitioner scheme in Norway.**

The Norwegian health care system is based on the principles of universal access, decentralization, and continuity of care. Since 2001, all Norwegian citizens may sign up with (and change, if desired) a general practitioner (GP), and 99% have chosen to do so. The scheme is rigged to offer continuity and close follow-up. It is financed by taxation, together with income-related employee and employer contributions and out-of-pocket payments (copayments). Private medical insurance is limited. GPs act as coordinators of municipal services and gatekeepers to specialized care. On average, a GP has a list of approximately 1050 patients. The Norwegian GP scheme is highly valued by patients [23].

### Data Analysis

We applied reflexive thematic analysis to the free-text material. This involved dynamic, repeated movement through 6 analytic phases. Initial familiarization with the data set (phase 1) and the generation of preliminary codes (phase 2) were primarily undertaken by BN and TMJ, who are both active GPs with experience from VC research [7]. Identification and review of candidates and final themes (phases 3-5), selection of relevant data extracts, and writing up (phase 6) were performed together with LOG with input from the whole author team. The analysis was initially inductively oriented but later came to involve a deductive mode transitioning from a semantic to a more latent orientation [25,26]. In particular, we concluded that our central themes all had a direct or indirect relationship with the phenomenon *trust* [27,28], as outlined in the discussion.

### Ethical Considerations

Ethical considerations were included in all phases of the survey. Participating GPs were informed that participation was voluntary and anonymous. We did not elicit sensitive information or demographic characteristics that could reveal the identity of the GPs. For the evaluated VCs, we did not elicit patients’ age, sex, specific diagnoses, or other sensitive or person-related information. Distribution of the survey to GPs’ email addresses was handled by an independent party (Norwegian Health Informatics). No linkage key was established, and participants’ IP numbers were not accessible to any party. Further approvals were thereby not required, according to Norwegian health research legislation, verified by the Norwegian Centre for Research Data (NSD).

## Results

The analysis of the free text answers regarding the use of VCs by Norwegian GPs resulted in a number of preliminary themes and 4 final themes (Table 1).

**Table 1:** Overview of preliminary and final themes.

Preliminary themes	Final themes
Continuity or relation Known patient or issue	Previous relation and contact enhance suitability
Establishing effective trajectories Safer remote triage than telephone only Tailoring the trajectory Joint Consultations The new “home visit”	Experiences of new potentials: optimizing workflow
Clinical suitability for specified reasons for contact Infection control Administrative issues and sick leave as part of trajectory Mental health issues Establishing contact with hesitant/vulnerable patients	Experiences of new potentials: exploring specific areas of clinical suitability
Adapting to a new consultation style One-problem approach Degradation of communication quality Impact of physical absence	Leaving the physical gold standard—weighing benefits against downsides
Inequity—lack of digital competence Distractions and threatened confidentiality Erosion of the basic trustful alliance	Quality and safety in a wider perspective

### Previous Relation and Contact Enhance Suitability

Many GPs reported that VCs appear, in general, more suitable when they have previous knowledge of the patients or the presented problem. An existing relationship gives patients confidence in their GP and, at the same time, strengthens the GPs’ ability to evaluate the patient’s health literacy and clinical judgements.

As a long-term GP with a stable patient population […] I know them well and they know me. I know how they express themselves. I know their personalities and patterns of behavior. It is very important to trust the content of a video consultation, knowing what is behind. Not a snapshot, not a drop-in.

### Experiences of New Potentials

#### Optimizing Workflow

Several of the GPs mainly compared VCs to telephone consultations and stated that VCs provide a welcome opportunity to gain a better understanding and overview of the patients’ health status and level of symptom severity. In many instances where only the telephone would otherwise have been the available modality, the quality of triage was enhanced by use of VCs.

…children who are described as very ill, but crawl effortlessly around on the mother’s lap, up and down on the sofa.

A number of GPs described how VCs enable them to establish tailored and more effective clinical trajectories and thereby optimize the clinical workflow. Examples were using VCs as the first point of contact to gain an initial, brief overview of the problem, or for follow-ups after a previous in-person consultation.

Video is suitable where problems have been clarified after a previous examination and initiation of treatment, where one mainly needs an anamnestic follow-up - it saves time and resources.

While in-person consultations tend to consume more time when the patient “is first there,” VCs are more targeted and freer from diversions and distractions.

I have experienced the video consultations as more targeted…

Many (patients) […] keep coming back to the physical things (“spots, scars, chronical problems, asymmetric skin folds, etc.”) during physical consultations. This often interrupts/disrupts a good dialogue/conversation between doctor and patient in relation to what is really the problem.

Most GPs confirmed that VCs are suitable to provide advice, clarify and explain information (eg, reports from hospitals and results from blood tests), and conduct follow-up consultations. Overall, VCs were described as informative, effective, easy, convenient, and safe. They were also deemed to increase patient engagement when compared to telephone consultations. Overall, VCs seem to stimulate a more optimal workflow, including follow-up of drug prescriptions.

I asses if patients find their medications and check what the medication lists should be, they can show what they have at home, many situations where patients are more relaxed...

An unusually experienced VC user reported having developed knowledge of the use and limitations of VCs at a high level.

I have been a pilot GP for VC for over 4 years, and it works perfectly even for patients you don't know from before. It's important to know your own limitations and clarify with the patient during and at the end what the next step is, in terms of advice, treatment, if the condition is not getting better or there is a need for follow-up and questions.

Some GPs saw for the first time a potential in the use of VCs to have joint consultations with more than 1 participant, including other health care professionals.

I have had some very good VCs in collaboration with home nursing. For example, I had one patient with infection in the jaw with swelling. It was a complex situation. The patient had to have a CRP test - so the home nurse came for helping to take a blood test and giving advice.

The GPs appreciated the contextual insight that VCs provided into the patients’ homes, which improved their evaluation of their physical and psychological climate.

It is nice to use video to keep in touch with families with young children. You get insight through a glimpse into how the family works and feels like at home. The children quickly get familiar with the camera.

In VCs, family members and other trusted listeners can support patients to better understand the GP’s advice (eg, how to adhere to medications). This may result in better communication, strengthen the patient-provider relationship, and increase mutual trust. Participating GPs pointed out how the responsibility for patient safety shifted toward the patient as they, for example, communicate more directly and protect the place from which the consultation takes place.

#### Exploring Specific Areas of Clinical Suitability

Many free-text responses concerned the use of VCs to evaluate specific clinical issues. In the context of the pandemic, many GPs evidently found it beneficial to triage, diagnose, and treat infectious diseases via video, thus avoiding contagion of COVID-19 or other diseases.

Reduce the risk of infection with infectious diseases, e.g., gastroenteritis or flu, but more difficult to assess severity on video.

VCs were also deemed suitable for extension of sick leave, to deliver medical certificates to the Norwegian Labor and Welfare Administration, absence certificates for school, and other sick leave reports (eg, disabled parking and taxi cards).

Much sick leave follow-up that does not require a physical examination is much better on video. It saves a lot of time for both doctor and patient.

During the lockdown in early 2020, VCs with patients with mental health problems and psychiatric conditions were apparently considered safe, at least as long as the GP had an established relationship with the patient. VCs appeared particularly useful for evaluating the patients’ level of anxiety as well as for psychological supportive therapy.

VCs are suitable for follow-up of psychiatric conditions where a good connection has already been established, such as follow-up of homework in cognitive therapy for anxiety and depression.

The same applied to follow-up evaluations of patients with complex and severe psychiatric conditions. Interestingly, several GPs reported that VCs can be helpful for building relationships with vulnerable patients who otherwise might be hesitant to seek help from their GP or open up on sensitive issues. The examples included relationally vulnerable patients such as young people with low self-confidence who might perceive an in-person consultation as overwhelming, socially withdrawn individuals who struggle with social anxiety and distrust, and relatively resourceful persons in acute despair.

[…] perhaps for patients with psychological problems who find it easier to open up when the physical proximity is not too close and for patients who would not otherwise go to the doctor.

### Leaving the Physical Gold Standard: Weighing Benefits Against Downsides

Compared to in-person consultations, the GPs reported how patients seem to present fewer questions and clinical issues in VCs, as described above. GPs described this as a “one problem only” phenomenon. On the one hand, this can be beneficial in the sense that the patients stay focused on the most essential clinical problem.

More effective with video, fewer problems, and less small talk.

Some GPs were, however, concerned that they could lose valuable communicative information during VCs, overlooking why the patient really sought help. VCs easily led to “closed” questions that do not further stimulate patients to describe their problem. To compensate, some GPs started to put in extra effort into investigating the patients’ reasons for contact.

Many GPs reported that they were afraid of not detecting signs of serious illness. The web-based communication climate implies degradation of valuable nonverbal signals that are more evidently present in in-person consultations: active listening, picking up signals, and validating patients’ presence.

The communication can feel strained and unnatural [...] One might easier lose something along the way in communication, it becomes a little less in depth - the small, subtle changes in attitude, tone, expression, pauses, become different.

GPs claimed that the extensive use of VCs could lead to delayed requests. Due to the absence of clinical examinations or in-person appointments during the pandemic, VCs could sometimes cause significant professional insecurity and force the GPs to make medical decisions based on “gut feelings.”

We may be tempted to rely on poorer clinical examinations on the screen that we could interpret differently in 3D [referring to an in-person consultation] or when we can use more senses. We are also not trained to make such assessments; our entire education is based on the fact that we can examine the patient in the same room.

There is a lack of any opportunity to assess body language and skin color, many small things which wake up the doctor’s ‘gut feeling’ in a physical consultation and which are difficult to put into words (direct gaze, posture, walking pattern, the patient’s non-verbal reactions, for example that he stiffens after a particular question or claim).

Some participants described that VCs might cause clinical uncertainty and lower the threshold for prescriptions and sick leave.

### Quality and Safety in a Wider Perspective

A few GPs expressed concerns about technical problems associated with VCs, mostly user errors on the patients’ side. Some GPs were also worried that VCs may create inequity, as resourceful, technology-experienced patients are likely to fill available slots for VCs. During the lockdown, when access to in-person consultations was limited, this could result in an increased risk for patient safety.

Not everyone is able to use VCs - vulnerable patients can go under the radar.

Privacy and confidentiality in the doctor-patient encounter was also an issue in the free-text responses. VCs can be disturbed or surveilled by listeners (ie, family members) on the patient’s side with or without making their presence known to the GP.

Other people in the room disturb, want to participate in the conversation, or have their own consultation [referring to family members who also have questions]. It can also be challenging regarding confidentiality if patients conduct video from wherever…!

[In the] home situation, it can be difficult to tell personal things - for example when a doctor wants to ask about sexual abuse, violence, and previous trauma experiences in relation to issues the patient raises […] You also cannot know whether the patient’s communication with the doctor is monitored by relatives and thus controlled.

Some GPs were worried that VCs could undermine the experience of a reciprocal trustful relationship characterized by openness, small signs of goodwill, and even humor, which may arise more naturally in an in-person encounter. Others referred to erosion of the therapeutic relationship, indirectly indicating loss of quality, which might even have implications for patients’ safety in a longer perspective.

## Discussion

### Principal Results

Based on comprehensive free-text material provided by 657 regular GPs in Norway during the COVID-19 lockdown in 2020, this study found VCs most suitable for consultations with previously known patients and follow-up evaluation of previously known health problems. GPs reported that VCs may facilitate the tailoring of effective clinical trajectories, including initial triage to determine the eventual need for further investigations. For better or worse, VCs tended to concern “one problem only.” Many GPs described the communication style on video as minimalistic and associated this with a certain loss of quality. On the positive side, VCs could provide new and valuable insight into the patients’ psychosocial circumstances and revealed interesting possibilities of establishing new relationships with patients who might otherwise not seek help.

### Comparison With Prior Work

#### Preexisting Relationships and Contact Enhance Suitability

Several studies attempted to clarify for which clinical issues VCs are most suitable [4,5,17,18,29]. In contrast, we argue that this attempt appears difficult and rather unrealistic to determine. Instead, the suitability of VCs seems to vary considerably, depending on whether a preexisting relationship between a patient and a doctor or a medical plan has already been established [4]. The clinical setting and relationship may, in other words, be more decisive than the specific reason for contact. From the perspective of trust, GPs associate preexisting knowledge with more effective and professionally satisfactory clinical interaction. Among other things, this enables GPs to better evaluate the individual patient’s health literacy and capacity to present health-related issues in a manner that can be safely evaluated and handled at distance.

#### New Potentials Revealed

Preexisting evidence suggests that some patients may experience difficulties in establishing a doctor-patient relationship when using VCs, feeling rushed and having trouble with taking turns in communicating [20,30]. In contrast, some GPs in this study reported having established relationships with patients who, for different reasons (social withdrawal, anxiety, lack of trust in health personnel, and practical hindrances), might otherwise have been reluctant to attend an in-person consultation. In such instances, VCs stand out as an alternative to not receiving health care at all [31,32] and may lower the threshold to in-person consultations after establishing an initial relationship.

Our study aligns with previous studies that found VCs suitable for triaging and assessing the need for further follow-up [1,11]. In addition, we found that VCs may provide valuable insight into patients’ life circumstances and available support in the home setting. Such contextual insight may also raise awareness of socioeconomical stressors in patients’ lives (eg, crowded housing, strained relationships, and challenges associated with child upbringing).

#### Demarcated Communication Is a Double-Edged Sword

In line with some previous studies, our informants reported a lower amount and richness of information exchanged in VCs than those in in-person consultations [5,33,34]. The fact that patients tend to discuss one problem only in VCs may entail the increased risk that GPs miss informative symptoms and signs that might not appear particularly relevant to the patient [35]. In addition, some GPs reported a significant absence of small talk in VCs compared to in-person encounters. At first glance, communicational minimalism might be interpreted as a sign of clinical effectiveness. However, research indicates that friendly, informal exchanges about neutral everyday matters represent a social bonding ritual that supports reciprocal goodwill [36]. In other settings, this has been shown to improve clinical communication and increase patients’ level of comfort and satisfaction [37,38].

#### Shifts of Responsibility and Challenges to Maintaining Trust

The GPs in this study noted that patients need to take wider responsibility in VCs than in in-person consultations. One aspect concerns health literacy and reflects the patients’ ability to present their reason for contact in a concise and comprehensible way for the GP via a digital interface. Furthermore, the VC modality assigns patients the responsibility of deciding the time and space for undisturbed and confidential communication, either with the patient alone or in the presence of welcome and trusted persons who can support and assist. Previous studies, however, have shown that the presence of dominant personalities on the patient’s side may represent a threat to patient integrity and safety and make it difficult for GPs to clearly identify and act upon [39-43]. This challenge was also reported by some GPs in our study.

The fact that VCs shift certain aspects of responsibility toward the patient highlights the value of preexisting reciprocal knowledge and trust. Patients’ trust in their doctors is typically rooted in expectations linked to the professional’s role, but concrete experiences of abilities, competence, and goodwill over time may be needed to solidify the relationship. Previous studies indicated that in-person consultations are best suited to establish durable clinical trust and are likely to endure under less optimal conditions [44-46].

### Strengths and Limitations

This nationwide web-based cross-sectional survey recruited a high number of GPs in Norway, thus increasing the external validity of the results [4]. It was conducted early during the first COVID-19 lockdown, when VCs reached an all-time peak and were used to handle an unprecedented range of conditions. While clearly limiting transferability to a normalized situation, the lockdown situation and a high number of novice VC users can be seen as an ultimate stress test regarding both potentials and shortcomings that could never have been ethically defendable under normal circumstances. While the free-text format does not provide in-depth information in line with interactive qualitative interviews, many of the answers contained thoughtful descriptions and reflections concerning GPs’ experiences with VCs. Thematic analysis was considered appropriate to identify patterned meaning across the material and place the findings in a wider context. Finally, the collaboration between experienced GPs and academics with varied backgrounds was deemed fruitful.

### Conclusions

This comprehensive free text-based analysis of GPs’ experiences of VCs during the COVID-19 lockdown revealed new potentials. Strategically planned VCs may facilitate effective tailoring of clinical trajectories, enhance the GPs’ insight in their patients’ relational and socioeconomical resources and vulnerabilities, and even facilitate relationship-building with patients in need of care who might otherwise be reluctant to seek help. VCs seem nevertheless most suitable for consultations with previously known patients. The study also discovered risky pitfalls. A demarcated communication style and a “one problem approach” may entail effectiveness in the short run. However, overreliance on VCs may, in a longer perspective, undermine the establishment and maintenance of relational trust, with a negative impact on quality of care and, ultimately, even patient safety.

### Implications

This study was conducted in a particularly stressful context, namely the COVID-19 lockdown. Collective GP experiences with the rapid, large-scale adoption of VCs can, however, be of interest also under less dramatic societal circumstances, particularly in comparable health care systems that offer some degree of continuity of care. GPs who lack the experience of VCs might use these results as a guide to identify and explore potentials and pitfalls associated with the uptake of VCs in their own local environment. Future research might also focus on comparing GPs’ experiences with different forms of remote consultations (video, SMS text messaging–based, and telephone consultations) as well as compare experiences from GPs with those from patients.

## Abbreviations

GP: general practitioner
VC: video consultation

## Data Availability

The data sets generated and analyzed during the current study are not publicly available due to Norwegian regulations, but access to the entire data material is available on reasonable request to the corresponding author.
